# Редкий случай гормонально-активной гонадотропиномы, ассоциированной со вторичным эритроцитозом, у мужчины в пожилом возрасте

**DOI:** 10.14341/probl12758

**Published:** 2021-06-13

**Authors:** Е. О. Мамедова, Л. С. Селиванова, К. А. Потапова, С. А. Бурякина, В. Н. Азизян, А. Ю. Григорьев, Ж. Е. Белая

**Affiliations:** Национальный медицинский исследовательский центр эндокринологии; Национальный медицинский исследовательский центр эндокринологии; Национальный медицинский исследовательский центр эндокринологии; Национальный медицинский исследовательский центр эндокринологии; Национальный медицинский исследовательский центр эндокринологии; Национальный медицинский исследовательский центр эндокринологии; Национальный медицинский исследовательский центр эндокринологии

**Keywords:** аденома гипофиза, гонадотропинома, ЛГ, ФСГ, гиперандрогения, эритроцитоз

## Abstract

Гормонально-активные гонадотропиномы — это редкие опухоли гипофиза, секретирующие один или два гонадотропных гормона (фолликулостимулирующий гормон (ФСГ) и/или лютеинизирующий гормон (ЛГ)), которые обладают биологической активностью. В большинстве случаев гонадотропиномы являются «молчащими» и составляют более половины гормонально-неактивных аденом гипофиза. В статье представлено описание редкого клинического наблюдения: ЛГ/ФСГ-секретирующей макроаденомы гипофиза с развитием битемпоральной гемианопсии у 62-летнего мужчины. Пациенту была проведена трансназальная транссфеноидальная аденомэктомия, позволившая достичь ремиссии заболевания. Отличительной особенностью данного случая являлось наличие вторичного эритроцитоза, развившегося вследствие эндогенной гиперандрогении, что потребовало проведения процедур эксфузий крови с целью нормализации уровня гематокрита перед проведением оперативного вмешательства. Примечательно, что клинические признаки эритроцитоза у пациента были выявлены задолго до развития зрительных нарушений. Представленный клинический случай демонстрирует сложность ранней диагностики гормонально-активных гонадотропином.

## Abstract (en)

Functioning gonadotroph adenomas are rare pituitary tumors secreting one or two gonadotropins (follicle-stimulating hormone (FSH) and/or luteinizing hormone (LH)), which are hormonally active. In the majority of cases, gonadotroph tumors are endocrinologically “silent” and make up more than a half of non-functioning pituitary adenomas. In this article we describe a rare clinical case of LH/FSH-secreting pituitary macroadenoma with bitemporal hemianopsia in a 62-year-old man. The patient underwent transnasal transsphenoidal adenomectomy, leading to remission. The distinctive feature of this case is the presence of secondary erythrocytosis due to endogenous hyperandrogenism, which required several blood exfusions to normaliza the level of hematocrit before surgery. It is noteworthy that clinical signs of erythrocytosis were present long before visual impairment. This clinical case demonstrates difficulties in the early diagnosis of functioning gonadotroph adenomas.

## АКТУАЛЬНОСТЬ

Гормонально-активные гонадотропиномы — это редкие опухоли гипофиза, секретирующие один или два гонадотропных гормона (фолликулостимулирующий гормон (ФСГ) и/или лютеинизирующий гормон (ЛГ)), которые обладают биологической активностью [1]. Примечательно, что гораздо чаще гонадотропиномы являются «молчащими», то есть имеют положительное окрашивание к ФСГ и/или ЛГ при иммуногистохимическом (ИГХ) окрашивании, но при этом либо не секретируют эти гормоны, либо секретируют их в биологически неактивной форме (альфа-субъединица, бета-субъединица ФСГ или ЛГ) и, следовательно, не сопровождаются симптомами гормональной гиперсекреции [1–4]. В целом «молчащие» гонадотропиномы встречаются достаточно часто и составляют до 64% всех гормонально-неактивных аденом гипофиза [5], тогда как гормонально-активные гонадотропиномы описаны в единичных случаях или в небольших сериях клинических наблюдений.

Клинические проявления гормонально-активных гонадотропином зависят от пола и возраста. У детей эти опухоли вызывают преждевременное половое развитие [6–8]. У женщин репродуктивного возраста длительное воздействие ФСГ на яичники приводит к нарушению менструального цикла, бесплодию, кистозным изменениям в яичниках, синдрому гиперстимуляции яичников, возникновению хронической боли в тазовой или брюшной областях [9–15]. В постменопаузальном периоде на первое место в клинической картине заболевания выступают признаки, обусловленные масс-эффектом опухоли (хиазмальный синдром, головные боли, гипопитуитаризм). Высокий уровень гонадотропинов у женщин в постменопаузе не влияет на функцию яичников и продукцию эстрогенов, поэтому секреция биологически активных гормонов опухолью гипофиза не приводит к развитию синдрома гиперстимуляции яичников [1]. У мужчин отмечаются увеличение тестикул, снижение либидо и эректильная дисфункция, реже наблюдается изолированное снижение зрительных функций без признаков гипогонадизма [2][3][9][16]. При гиперсекреции ЛГ опухолью может отмечаться повышение уровня тестостерона в крови [3][17].

Основным методом лечения гонадотропином является транссфеноидальная аденомэктомия, позволяющая в ранние сроки достичь ремиссии заболевания [1]. В случае невозможности радикального удаления опухоли (инвазивный рост, прорастание в кавернозные синусы) или рецидива возможно рассмотрение вопроса о проведении лучевой терапии [1]. Медикаментозная терапия (аналоги соматостатина, агонисты дофамина, темозоламид) не является методом выбора лечения гонадотропином в связи с ограниченными данными о ее эффективности [1].

В данной статье представлено описание редкого клинического наблюдения: ЛГ/ФСГ-секретирующей макроаденомы гипофиза с развитием битемпоральной гемианопсии, вторичного эритроцитоза у 62-летнего мужчины, описаны особенности диагностического поиска и результаты проведенного лечения.

## ОПИСАНИЕ СЛУЧАЯ

Пациент Г., 62 лет, поступил в отделение нейроэндокринологии и остеопатий ФГБУ «НМИЦ эндокринологии» Минздрава России в сентябре 2020 г. с жалобами на выраженное снижение остроты зрения, «белую пелену» перед глазами, сужение полей зрения, периодическую головную боль.

Из анамнеза известно, что вышеописанные жалобы стали беспокоить пациента с декабря 2019 г. В феврале 2020 г. пациент самостоятельно обратился к офтальмологу по месту жительства, при осмотре выявлены частичная атрофия зрительных нервов, начальная катаракта обоих глаз; офтальмологом заподозрена патология гипофиза, рекомендовано выполнение магнитно-резонансной томографии (МРТ) головного мозга. Ранее, со слов пациента, при ежегодных профосмотрах (последний в 2019 г.) значимой патологии со стороны органов зрения не отмечалось. При МРТ головного мозга обнаружено объемное образование хиазмально-селлярной области размерами 44×34×43 мм. Для дальнейшего обследования и оперативного лечения пациент с направительным диагнозом «Неактивная аденома гипофиза» был госпитализирован в ФГБУ «НМИЦ эндокринологии» Минздрава России.

При первичном осмотре обращали на себя внимание инъекция сосудов склер, гиперемия лица, шеи, верхней половины туловища, ладоней; клинических признаков акромегалии, гиперкортицизма выявлено не было. Пациент гиперстенического телосложения, рост 174,4 см, масса тела при поступлении 102 кг (ИМТ=33,5 кг/м2). Распределение подкожной жировой клетчатки равномерное, отеков нет. Оволосение по мужскому типу, вторичные половые признаки развиты правильно. Печень при пальпации +1 см от края реберной дуги, край печени закруглен, уплотнен, безболезнен. В остальном по органам и системам без особенностей. Анамнез жизни и наследственность не отягощены; курение, употребление алкоголя отрицает.

По данным МРТ головного мозга от 17.09.2020 г. на серии сагиттальных, фронтальных и аксиальных томограмм, выполненных в режимах Т1- и Т2-взвешенных изображений (ВИ), в полости турецкого седла определяется образование с четкими ровными контурами, размерами: ширина 40 мм, высота 41 мм, переднезадний размер 33 мм. Структура его неоднородная за счет жидкостных включений. Образование распространяется супраселлярно с компрессией хиазмы зрительных нервов и дна третьего желудочка, прилежит к передним соединительным артериям, латерально в кавернозные синусы с циркулярным окружением кавернозных отделов внутренних сонных артерий (Knosp IV), прилежат к медиобазальным отделам височных долей и прямым извилинам. Кпереди образование пролабирует в основную пазуху, кзади — разрушает спинку турецкого седла и распространяется в предмостовую цистерну с умеренным ее сужением (рис. 1). Неизмененная ткань гипофиза, воронка не дифференцируются. По заключению: макроаденома гипофиза с супра-, инфра-, ретро-, параселлярным (D, S) распространением (Knosp IV).

**Figure:**
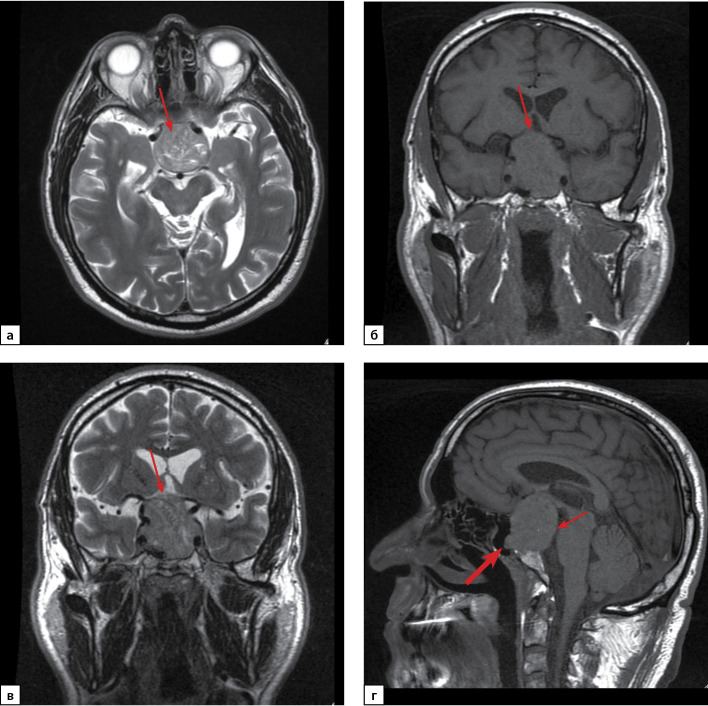
Рисунок 1. МРТ головного мозга пациента Г. Макроаденома гипофиза с супра-, инфра-, ретро-, параселлярным (D, S) распространением (Knosp IV):а) Т2-ВИ (взвешенное изображение), аксиальная проекция. Аденома гипофиза (стрелка); б) Т1-ВИ, корональная проекция; в) Т2-ВИ, корональная проекция. Компрессия хиазмы зрительных нервов (стрелка); г) Т1-ВИ, сагиттальная проекция. Распространение аденомы инфраселлярно в основную пазуху (толстая стрелка) и ретроселлярно в предмостовую цистерну (тонкая стрелка).

При осмотре офтальмологом выявлено: острота зрения OD=0,2 н/к, OS=до 0,8 эксцентрично; частичная атрофия зрительных нервов обоих глаз. В ходе периметрии выявлено сужение полей зрения (рис. 2А). По результатам гормональных исследований эндогенный гиперкортицизм и акромегалия были исключены (кортизол слюны вечером 5,11 нмоль/л (0,5–9,65), ИФР-1 104,6 нг/мл (16–245)), были подтверждены вторичный гипотиреоз и вторичная гиперпролактинемия (табл. 1, до операции). При исследовании гонадотропной функции определялось повышение уровня ФСГ при уровнях ЛГ и тестостерона на верхней границе нормы (табл. 1, до операции). В анализах крови также обращало на себя внимание значимое повышение уровней эритроцитов, гемоглобина, гематокрита (табл. 1, до операции), то есть выраженный эритроцитоз на фоне относительно нормальных значений остальных показателей гемограммы. По данным биохимического анализа крови уровень креатинина составил 117,2 мкмоль/л (рСКФ-EPI 58 мл/мин/1,73 м2), имелись признаки гиперурикемии, дислипидемии, в остальном — без значимых клинических отклонений. Примечательно, что в последние 5–6 лет пациент стал обращать внимание на покраснение лица, шеи, верхней половины туловища, однако, несмотря на ежегодные профилактические обследования, о наличии эритроцитоза пациент осведомлен не был.

**Figure:**
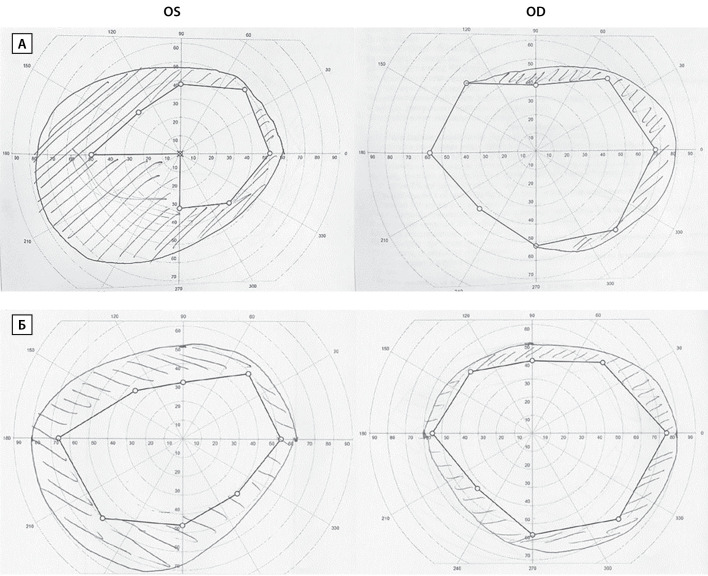
Рисунок 2. Результаты периметрии до операции (А, верхний ряд) и через 6 месяцев после операции (Б, нижний ряд). OS — левый глаз, OD — правый глаз.

Было проведено ультразвуковое исследование (УЗИ) органов мошонки от 16.09.2020 г.: объем правого яичка составил 22 мл, левого яичка — 20,7 мл. При УЗИ органов брюшной полости от 21.09.2020 г. выявлены эхо-признаки гепатомегалии (толщина правой доли 13,4 см, левой доли 6,2 см), жирового гепатоза, хронического калькулезного холецистита, селезенка без особенностей, с ровными четкими контурами, размерами 10,1×5,2 см.

Таким образом, на основании лабораторных исследований (высокие показатели ЛГ, ФСГ, тестостерона, вторичный эритроцитоз), верификации по данным МРТ объемного образования гипофиза у пациента было заподозрено наличие ЛГ/ФСГ-секретирующей макроаденомы гипофиза.

Учитывая наличие гонадотропин-секретирующей макроаденомы гипофиза с компрессией хиазмы, оптимальным методом лечения было выбрано нейрохирургическое вмешательство. В рамках подготовки к оперативному лечению с целью снижения риска тромбообразования пациенту проводились эксфузии цельной крови с замещением изъятого объема физиологическим раствором натрия хлорида (выполнено 3 процедуры) с достижением целевых значений показателей периферической крови (Hb до 150–160 г/л, Ht не выше 51%). В сентябре 2020 г. на базе ФГБУ «НМИЦ эндокринологии» Минздрава России пациенту выполнено трансназальное транссфеноидальное удаление макроаденомы гипофиза. Интраоперационно нейрохирургами обнаружена опухоль желто-коричневого цвета, умеренно плотной консистенции, которая была полностью удалена. По данным гистологического исследования — базофильная аденома гипофиза солидно-альвеолярного строения (рис. 3). При ИГХ-исследовании подтверждена экспрессия ЛГ в 80% опухолевых клеток (рис. 4) и ФСГ (рис. 5) — в 30% опухолевых клеток; выявлена слабая реакция к рецепторам соматостатина 2А (рис. 6) и 5 типов (рис. 7) (3 балла по IRS). Индекс Ki-67 составил 3% (рис. 8). Таким образом, был верифицирован диагноз ЛГ/ФСГ-секретирующей аденомы гипофиза.

**Figure:**
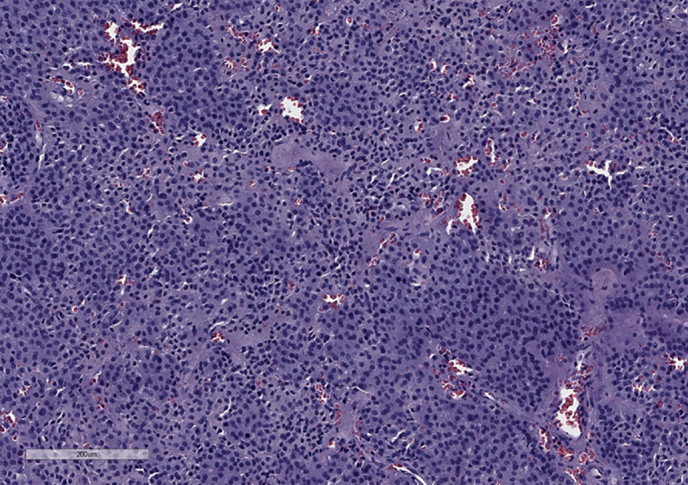
Рисунок 3. Микроскопическое строение опухоли. Окраска гематоксилином и эозином. Увеличение х100.

**Figure:**
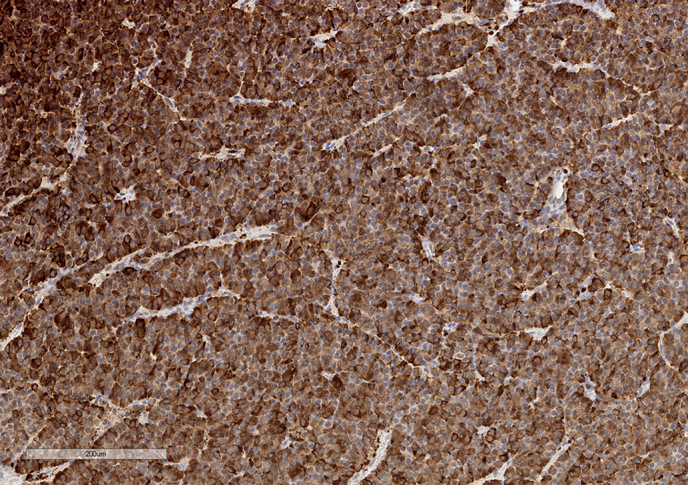
Рисунок 4. Экспрессия лютеинизирующего гормона опухолевыми клетками. Увеличение х100.

**Figure:**
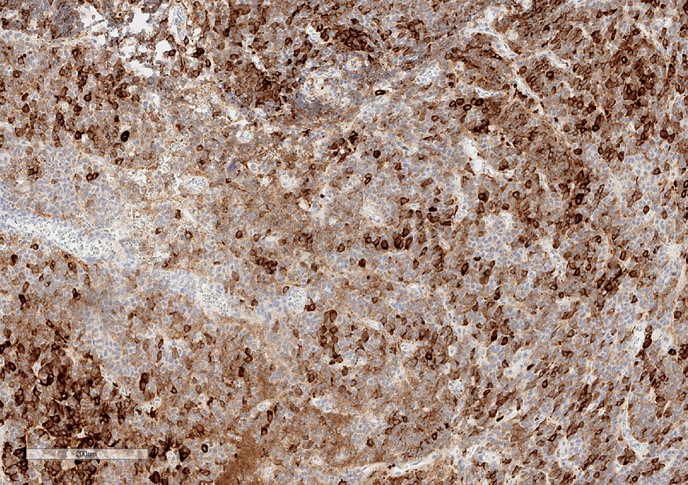
Рисунок 5. Экспрессия фолликулостимулирующего гормона опухолевыми клетками. Увеличение х100.

**Figure:**
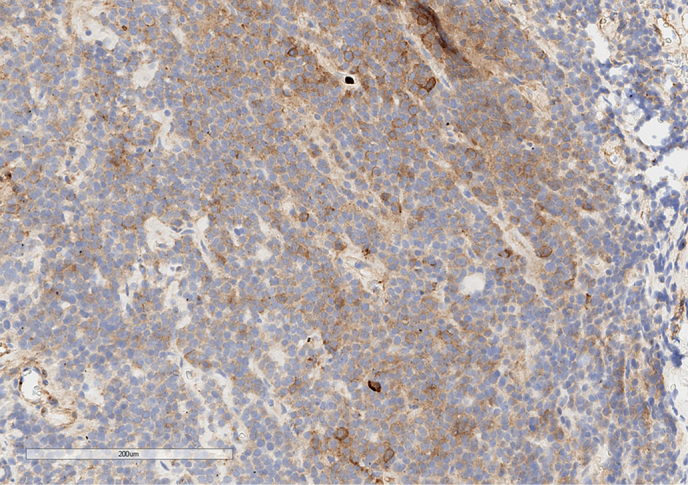
Рисунок 6. Экспрессия SSTR2A опухолевыми клетками. Увеличение х100.

**Figure:**
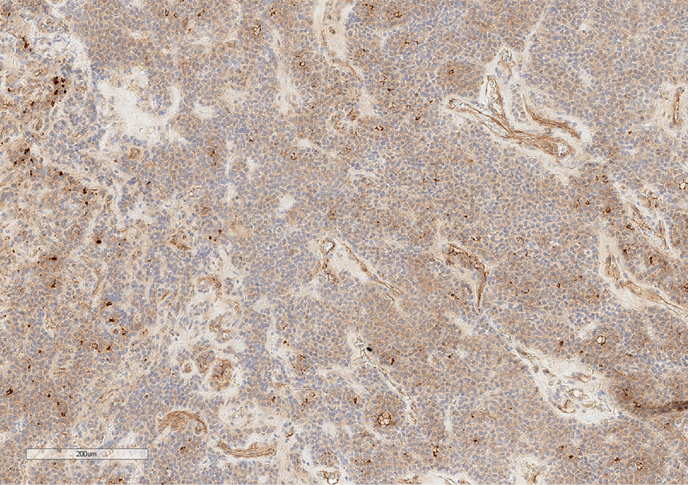
Рисунок 7. Экспрессия SSTR5 опухолевыми клетками.Увеличение х100.

**Figure:**
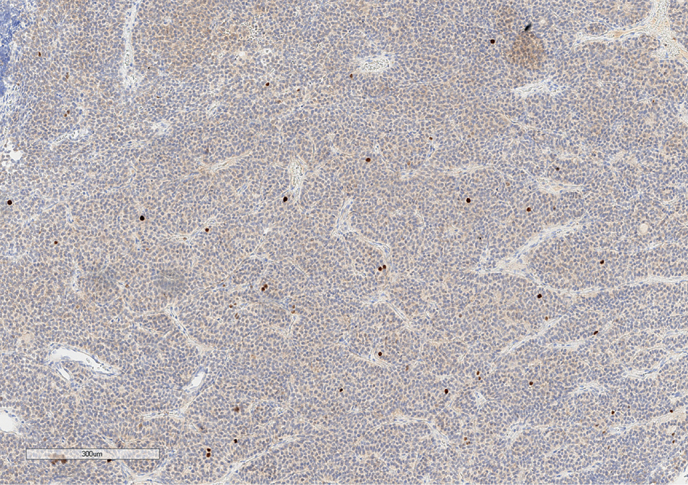
Рисунок 8. Индекс пролиферативной активности опухоли (по экспрессии Ki-67).

В раннем послеоперационном периоде отмечалось развитие вторичной надпочечниковой недостаточности (кортизол крови утром 175,6 нмоль/л; табл. 1, после операции), в связи с чем была проведена заместительная терапия гидрокортизоном. С целью коррекции вторичного гипотиреоза (табл. 1, после операции) последовательно было начато лечение левотироксином натрия в начальной дозе 50 мкг. Медикаментозную коррекцию вторичного гипогонадизма (табл. 1, после операции) рекомендовано было начать препаратами геля тестостерона после проведения УЗИ предстательной железы. В первую неделю после оперативного вмешательства отмечалась тенденция к гипонатриемии (до 134 ммоль/л), однако на фоне соблюдения питьевого режима (ограничение жидкости до 1 л/сут с постепенным расширением до 1,5–2 л/сут) уровень натрия нормализовался. Показатели АД, ЧСС за время наблюдения находились в пределах целевых значений. Пациент был выписан на 13-е сутки после оперативного вмешательства в удовлетворительном состоянии. Через полгода после операции сохранялась ремиссия заболевания: по данным лабораторного обследования сохранялись гипогонадотропный гипогонадизм, а также вторичная надпочечниковая недостаточность и вторичный гипотиреоз (табл. 1, 6 мес после операции). Пациенту был рекомендован прием гидрокортизона, левотироксина, препаратов тестостерона. При осмотре офтальмологом: острота зрения OD=0,4, OS=0,8; частичная атрофия зрительных нервов обоих глаз (OS>OD), положительная динамика полей зрения по данным периметрии (рис. 2Б). По данным контрольной МРТ головного мозга от 22.03.2021 г. в проекции турецкого седла, в кавернозных синусах, в пазухах основной кости имелось неоднородно накапливающее контрастный препарат кистозно-солидное объемное образование неоднородной структуры (содержит кисту размерами 11×16×13 мм), размерами: вертикальный — 20 мм, поперечный — 27 мм, переднезадний — 25 мм; ткань гипофиза нечетко дифференцировалась по правому контуру образования, воронка отклонена вправо. По заключению — состояние после удаления гигантской аденомы гипофиза; объемное образование селлярной области с инфра- и параселлярным (D, S Knosp III) распространением. Учитывая ремиссию заболевания по данным лабораторного исследования, выявленные на МРТ данные были расценены как послеоперационные изменения, рекомендован динамический контроль МРТ.

**Table:** Таблица 1. Данные лабораторных обследований до и после операции

Исследуемый параметр, единицы измерения	До операции	После операции	Через 6 мес после операции	Референсный диапазон
Общеклинический анализ крови
Эритроциты, кл/л	6,66×1012	5,15×1012	4,71×1012	4,3-5,8×1012
Средний объем эритроцитов, MCV, фл	85,1	87,2	83,9	82–98
Гемоглобин, г/л	196	152	137	132–172
Гематокрит, %	56,7	44,9	39,5	40–51
Лейкоциты, кл./л	7,38×109	8,52×109	4,54×109	3,9–10×109
Тромбоциты, кл./л	207×109	249×109	207×109	148–339×109
СОЭ, мм/ч	9	19	18	2–20
Гормональный анализ крови и мочи
ЛГ, Ед/л	10,3	0,513	0,216	2,5–11
ФСГ, Ед/л	22,5	4,19	0,87	1,6–9,7
Тестостерон, нмоль/л	28	0,62	0,271	11–28,2
ТТГ, мМЕ/л	0,795	0,088	1,071	0,25–3,5
Т4св, пмоль/л	6,95	5,78	7,09	9–19
ИФР-1, нг/мл	104,6	-	52,36	16–245
Пролактин, мЕд/л	562,1	-	178,1	78–380
Кортизол крови утром, нмоль/л	293,7	175,6	237,3	171–536
Свободный кортизол в суточной моче, нмоль/сут	-	-	52,7	100–379

## ОБСУЖДЕНИЕ

Представленный клинический случай демонстрирует сложность диагностики гормонально-активных гонадотропином. Особенностью этого случая являлась сочетанная гиперсекреция ФСГ и ЛГ, при этом преобладала секреция ЛГ опухолью, что привело к развитию гиперандрогении. Среди описанных в литературе гормонально-активных гонадотропином у мужчин (единичные случаи или небольшие серии случаев) в основном выявлялась гиперсекреция ФСГ опухолью, что приводило к увеличению яичек (при этом уровни ЛГ и тестостерона были низкими или нормальными); подавляющее большинство опухолей были макроаденомами, приводившими к развитию зрительных нарушений [2][4][8][9][18–20]. В ряде случаев описана сочетанная гиперсекреция ФСГ и ЛГ [3][16][17][21–24]. В единичных описанных случаях опухоль секретировала только ЛГ [25][26]. Примечательно, что в большинстве случаев сочетанной гиперсекреции ФСГ и ЛГ, приводившей к повышению уровня тестостерона, клинических признаков гиперандрогении не выявлялось [3][16][21][23]. В других случаях клинические признаки гиперандрогении включали: повышение сперматогенеза [22], усиление либидо [24], эритроцитоз [17]. Также примечательно, что в некоторых случаях ФСГ/ЛГ-секретирующих аденом, при подтвержденном повышении уровней ЛГ и тестостерона при лабораторном обследовании, при ИГХ-исследовании удаленной опухоли экспрессия ЛГ отсутствовала [3][24].

Основным клиническим проявлением гиперандрогении в описываемом нами случае было развитие вторичного эритроцитоза, который, по анамнестическим данным, возник за несколько лет до развития зрительных нарушений. Основные причины развития вторичных эритроцитозов представлены в таблице 2. В существующих рекомендациях по дифференциальной диагностике эритроцитоза избыток тестостерона в основном рассматривается только при передозировке препаратов тестостерона, тогда как вероятность развития эндогенной гиперандрогении не рассматривается [27][28]. Кроме того, в представленном нами случае уровень тестостерона находился в районе верхней границы референсного интервала, и о его избытке свидетельствовали лишь «неподавленные» уровни ЛГ и ФСГ у пациента с макроаденомой гипофиза, тогда как при экзогенной гиперандрогении эти показатели были бы снижены. Учитывая многообразие причин вторичного эритроцитоза, возможно предположить, что вероятность обнаружения гонадотропиномы на момент возникновения эритроцитоза в представленном случае была бы низка.

**Table:** Таблица 2. Причины эритроцитоза (адаптировано из [27] и [28])

Первичный эритроцитоз
Истинная полицитемия
Вторичный эритроцитоз
Генерализованная гипоксия
Курение
Отравление СО
Заболевания легких
Обструктивное апноэ сна
Врожденный порок сердца (шунт справа налево)
Проживание на большой высоте
Локальная гипоксия почки
Стеноз почечной артерии
Гидронефроз
Поликистоз почек
Ассоциированный с приемом лекарств
Тестостерон
Эритропоэтин
Патологическая продукция эритропоэтина опухолями
Гепатоцеллюлярная карцинома
Почечноклеточный рак
Гемангиобластома мозжечка
Рак околощитовидной железы
Лейомомы матки
Феохромоцитома
Менингиома

Хирургическое лечение является методом выбора лечения гормонально-активных гонадотропином, поскольку позволяет устранить масс-эффекты опухоли (в первую очередь устранить воздействие на хиазму) и достичь быстрой нормализации гормональных показателей [1]. Убедительных данных об эффективности медикаментозного лечения или лучевой терапии в литературе нет [1]. В представленном нами случае после оперативного удаления опухоли развилась ремиссия заболевания, однако сохранялся гипопитуитаризм и не в полном объеме восстановилась зрительная функция.

Таким образом, в представленном клиническом случае, как и в большинстве описанных ранее в литературе случаев, гормонально-активная гонадотропинома была диагностирована лишь на стадии зрительных нарушений при появлении масс-эффекта опухоли. Вопрос о возможной ранней диагностике таких опухолей в настоящее время не решен.

## ЗАКЛЮЧЕНИЕ

В отличие от других гормонально-активных аденом гипофиза, которые имеют характерные клинические проявления, симптомы гонадотропин-продуцирующих аденом часто ускользают от обнаружения до тех пор, пока они не станут макроаденомами, вызывающими головную боль, нарушения полей зрения и гипопитуитаризм. Также необходимо помнить, что вторичный эритроцитоз у мужчин может быть признаком гиперандрогении, одной из причин которой могут являться ЛГ-секретирующие гонадотропиномы.
